# Are there changes in the nutritional status of children of *Oportunidades* families in rural Chiapas, Mexico? A cohort prospective study

**DOI:** 10.1186/s41043-015-0038-5

**Published:** 2016-01-16

**Authors:** Esmeralda García-Parra, Héctor Ochoa-Díaz-López, Rosario García-Miranda, Laura Moreno-Altamirano, Roberto Solís-Hernández, Raúl Molina-Salazar

**Affiliations:** 1 Health Department, El Colegio de la Frontera Sur, Carretera Panamericana y Periférico Sur s/n C.P. 29290, Barrio de María Auxiliadora, San Cristóbal de las Casas, Chiapas Mexico; 2 Public Health Department, Faculty of Medicine, Universidad Nacional Autónoma de México, Circuito Interior, Ciudad Universitaria, Av. Universidad 3000, CP 04510 Mexico City, Mexico; 3 Department of Economics, Universidad Autónoma Metropolitana—Iztapalapa, Av. San Rafael Atlixco 186, Col. Vicentina C.P. 09340 Delegación Iztapalapa, Mexico City, Mexico

**Keywords:** Child malnutrition, Stunting, Social programs, Poverty, *Oportunidades / Prospera*, Chiapas, Mexico

## Abstract

**Background:**

In Mexico, despite that the fact that several social programs have been implemented, chronic undernutrition is still a public health problem affecting 1.5 million children of <5 years. Chiapas ranks first in underweight and stunting at national level with a stunting prevalence of 31.4 % whereas for its rural population is 44.2 %. The purpose of this paper is to determine if the nutritional status of a cohort of children living in poor rural communities under *Oportunidades* has changed. We were interested in assessing the nutrition evolution of the children who were initially diagnosed as stunted and of those who were diagnosed as normal. Oportunidades is an anti-poverty program of the Mexican government consisting mainly in monetary transfers to the families living in alimentary poverty.

**Methods:**

A 9-year cohort prospective study was conducted with nutritional evaluations of 222 children. Anthropometric indices were constructed from measurements of weight, height, and age of the children whose nutritional status was classified following WHO standards.

**Results:**

The results showed that although these children were Oportunidades beneficiaries for 9 years and their families improved their living conditions, children still had a high prevalence of stunting (40.1 %) and 69.6 % had not recovered yet. Children who were initially diagnosed with normal nutritional status and became stunted 2 years later had a higher risk (relative risk (RR) 5.69, 2.95–10.96) of continuing stunted at school age and adolescence.

**Conclusions:**

Oportunidades has not impacted, as expected, the nutritional status of the study population. These findings pose the question: Why has not the nutritional status of children improved, although the living conditions of their families have significantly improved? This might be the result of an adaptation process achieved through a decrease of growth velocity. It is important to make efforts to watch the growth of the children during their first 3 years of age, to focus on improving the diet of women at fertile age and pay special attention to environmental conditions to break the vicious cycle of malnutrition.

## Background

In the world, there are two billion people who have some deficiency of micronutrients and 1400 million are overweight, out of which 500 million are obese [1]. In spite of this, the malnutrition problems for short height (stunting) affect almost 200 million children under 5 years old in the world. In Latin American and Caribbean countries, the incidence data registers differences of up to 14 % points between the rural and the urban areas [2]. In Mexico, stunting affects 1.5 million children under 5 years of age [3]. Nowadays, Mexico faces the problem of malnutrition, which is expressed, on the one hand, with a great proportion of overweight and obese children, and on the other hand, infantile stunting and anemia [4]. Almost half of the children under five (27.5 %) who live in rural areas are stunted [5]. Between 2008 and 2010, the population living in poverty increased from 48.8 million to 52 million people (from 44.5 to 46.2 %), and ten million children (approximately 25 %) were unable to afford reasonable access to food, the so-called food poverty [2, 6].

The last National Health and Nutrition Survey (ENSANUT 2012) reported that the preschool and school populations of the southern region of Mexico still have a high prevalence of stunting (19.2 %) [5]. The most affected areas are the rural localities with a prevalence of 27.5 % compared with the national average of 13.6 %. Chiapas, Guerrero, and Oaxaca are among the less developed Mexican states, with the highest indexes of poverty, the poorest nutrition indicators, and the highest stunting prevalence [7]. Chiapas, where this study was conducted, comes first at national level in underweight and stunting prevalence, e.g., the prevalence of stunting among children under 5 years of age is 31.4 % at state level and for children living in rural area is 44.2 % [7].

Different social programs to combat poverty and undernutrition in Mexico have been implemented in the last four decades [8]. In 1997, Mexico launched a new incentive-based poverty reduction program, initially known as *Progresa*, in 2006 changed to *Oportunidades*, and since 2014 renamed as *Prospera*. Oportunidades focuses on enhancing the human capital of those living in extreme poverty [9]. According to the World Bank (2010), it is the principal anti-poverty program of the Mexican federal government and its aim is to break the intergenerational cycle of poverty by using cash transfers, targeted to the poorest families and conditioned to regular school attendance and family health clinic visits. In addition, households with young children are provided with a fortified food supplement (*Nutrisano*), and pregnant and breastfeeding mothers receive a fortified food (*Nutrivida*) [10].

The objective of this study was to identify the changes in the nutritional status of a prospective cohort, initiated in 2002, of children under 5 years from families affiliated to Oportunidades who live in poor rural communities of Chiapas. We were interested in assessing the nutrition evolution of the children who were initially diagnosed as stunted and of those who were diagnosed as normal.

## Methods

### Study area

Chiapas is a Mexican state, located in the south-eastern area of the country. According to the 2010 National Population Census, Chiapas has a population of 4,796,580 inhabitants out of which 51 % live in the rural area [11]. Chiapas is divided into 15 socioeconomic and geographic regions [12]. The results of the present research are based on the VII region called *De los Bosques*.

### Study population

This research was developed in four rural communities: *La Competencia*, *Ramos Cubilete*, *Rivera Domínguez*, and *El Jardín*. These municipalities were selected by a purposive sampling technique according to their level of marginalization [12], for being an indigenous area and a priority area for the Chiapas State Ministry of Health (SSA). The four communities were selected according to the following criteria: geographical access (two of difficult access and two near the head of the municipality), proportion of indigenous population, and health system that attends them, and the other two communities being served by the SSA and two under the health system of the federal government for the uninsured population operated by the Mexican Social Security Institute (IMSS), both institutions are the ones that operate the health component of Oportunidades.

The cohort study started with a first evaluation (baseline) conducted in 2002–2003 through a census of children under 5 years of age living in the four communities, registering a total of 407 children. Three hundred seventy-nine children out of the total were children from Oportunidades families, and the other 28 were non-Oportunidades children. For the second evaluation (2004–2005), to accomplish the study objectives, only the children under 5 years who still were receiving the benefits of Oportunidades, such as supplementary foods (*Nutrisano*), were included in the follow-up. Thus a total of 237 children met these criteria and were measured during the second evaluation; the other 142 children measured in the first evaluation did not fulfill the selection criteria for the second one. During the third evaluation (2010–2011), 15 children out of these 237 were lost (6.3 %), obtaining a total final sample of 222 children who participated in the three evaluations.

### Data source and study design

The data for the three evaluations were collected by household interview surveys under a prospective cohort design. During the baseline evaluation, three groups were defined according to the nutritional status of the children: stunted, normal, and high height. These three groups were followed up and evaluated twice more (2004–2005 and 2010–2011). The questionnaire used was designed and validated by the research team. The questionnaire included demographic (age of the mothers, gender, kinship, local language spoken) and socioeconomic (schooling, occupation, benefits from Oportunidades, access to social security, household conditions, and assets) information and nutritional status data. The fieldwork was done with the help of community health workers, local language speakers, who were not members of the communities under study.

### Anthropometric measures

Anthropometric measurements included weight and height data of all children living in the household included in the sample.

The anthropometric measurements were conducted by students (undergraduates in nutrition) and nutritionists who were trained according to the techniques described and recommended by the World Health Organization [13] with the help of community health workers for translating and interviewing. The fieldwork staff was trained and standardized according to conventional procedures [14, 15]. To measure the weight of children under five, in the first two evaluations, we used standardized spring loaded Salter scale. For children weighing more than 20 kg, we used standardized scale class III with a capacity for 150 kg. To measure the length of the under 2-year-olds, we used an acrylic infantometer of 85 cm, with a precision up to ±0.5 cm. To measure the height of those children older than 24 months of age, we used wall estadiometer (DAY designs BREU) of acrylic material with capacity of 2 m. During the third evaluation, we used the same scale and estadiometer as in the first and second evaluation. The calibration of the equipment was done by the nutritionist responsible of the fieldwork team. To calibrate the balances weights of 5, 10 and 20 kg were used. To calibrate the estadiometers, rods of 0.5, 1.0, and 1.5 m were used [16, 17].

The cutoff points used for classifying the nutritional status of the children were defined as follows: stunting (height-for-age): <−2 standard deviations (SD) from the reference median [18].

### Ethical approvals

According to Mexican health regulations, this study was considered as exempt from IRB review due to the non-invasive methods used. Informed consent was obtained verbally from all participants.

During the induction of the study in the study area, permissions for conducting fieldwork activities were obtained from the health community authorities at each locality. Before the administration of the questionnaires, a verbal informed consent explaining the purpose of the interview and giving assurances of the confidential use of the information was obtained from the head of each of the households visited. Those cases of children with nutritional problems were immediately referred to the nearest health center for their medical attention.

### Statistical analyses

In the data entry phase, the information was processed in SPSS version 15.0.1, while for the anthropometry data, according to the age of the children; we used WHO AnthroV.3.1.0 [19] for children of 0–60 months; and WHO AnthroPlus V.1.0.2 [20] for children between 5 to 13 years of age based on WHO tables of reference [18]. These tables were used to obtain *Z* values for the height/age index. For the analysis of differences between proportions, chi-square test and *F* test were used. We calculated crude and stratified (age and sex) relative risk (RR) with a 95 % confidence interval to assess the risk of continuing stunted (cases) in the third evaluation among the children under study by comparing stunted children (exposed group) *vs* normal and high height (non-exposed group) in the second evaluation. For this analysis, we used Stata/SE 10.0 for Windows, (2008).

## Results

### Living conditions of the study population

Table 1 describes the living conditions of the children’s families under study. We focused on analyzing changes in demographic and socioeconomic characteristics after 9 years from the first evaluation. In general, all the living conditions indicators have improved significantly. The educational level improved as the number of illiterates diminished 12.8 percentage points (from 35.2 to 22.4 %), the number of people without schooling decreased (from 31.7 to 22.5 %), while the mean years of schooling among people over 15 years old increased 1.45 years. Housing conditions also improved during the study period. For example, the number of households with dirty floors decreased significantly and the number of household with piped water has increased. Consumption of meats, utilized as income indicator, increased significantly during the 9-year period, as it is shown in Table 1.

**Table 1:** Changes in the living conditions of the participant’s families during follow-up period

Year of evaluation	2002–2003	2004–2005	2010–2011	*p* ^a^
Number of people	1093	1106	1060	
Mean age in years of the children’s mothers (SD)	27.31 (6.77)	29.43 (8.05)	36.83 (7.95)	*p* = 0.000
Children under 5 years old, global and by sex^b^				
Total	26.2 %	23 %	10.6 %	*p* = 0.000
Men	11.7 %	11.2 %	4.8 %	*p* = 0.000
Women	14.5 %	11.8 %	5.8 %	*p* = 0.000
Illiterate population over 15 years old	35.2 %	30.7 %	22.4 %	*p* = 0.000
No-schooling population over 15 years old	31.7 %	27.3 %	22.5 %	*p* = 0.000
Number of homes visited	159	157	157	
Homes with dirty floor	93.7 %	84.3 %	15.1 %	*p* = 0.000
Houses with electricity	93.1 %	97.5 %	100.0 %	*p* = 0.001
Homes with refrigerator	9.4 %	14.5 %	40.3 %	*p* = 0.000
Houses with TV	32.7 %	50.9 %	66.7 %	*p* = 0.000
Households with piped water	78.6 %	81.8 %	97.5 %	*p* = 0.000
Overcrowded housings	91.2 %	92.5 %	72.3 %	*p* = 0.000
Families who eat red meat once a month	23.3 %	30.8 %	40.3 %	*p* = 0.005
Average income from *Progresa-Oportunidade*s per person (USD)	$6.91	$8.91	$29.38	*p* = 0.000

^a^On a chi-square test for proportions and *F* test for averages

^b^In the first, second, and third evaluation, all children were under 5 years of age. In the second evaluation, only cohort children still under 5 years of age were included; the other children were their brothers and sisters who fulfill the criteria of being under 5 years of age. In the third evaluation, any children from the cohort were included for being older than 5 years of age

The age distribution of the cohort of children was as follows: 114 females of whom 56 were 0–23 months of age, 58 were 24–60 months; 108 males of whom 56 were 0–23 months of age and 52 were 24–60 months of age.

### Cohort analysis of the nutritional status of the children

During the first evaluation, children were classified in three categories according the height/age index. These categories were as follows: stunting children, normal children, and children with high height for their age. Children in each category were followed up and their nutritional status was assessed to ascertain how many of them recovered and how many got worse.

Table 2 describes the movement between the nutritional status categories of the prospective cohort of the children during the follow-up period. It also shows that after 9 years of the first evaluation, the stunting problem still persists. Within the initial group of children diagnosed as stunted (104), 76 % of these children were still stunted in the second evaluation and 69.6 % children were still in the same nutritional category in the third evaluation. Likewise, 110 children who were diagnosed as normal in the first evaluation in the second evaluation, 34.5 % of these children were identified as stunted and in the third evaluation 52.6 % children (out of 38) were still in this same condition. From the group of children diagnosed as normal the second evaluation (71), only 88.7 kept being diagnosed as normal during the third evaluation.

**Table 2:**
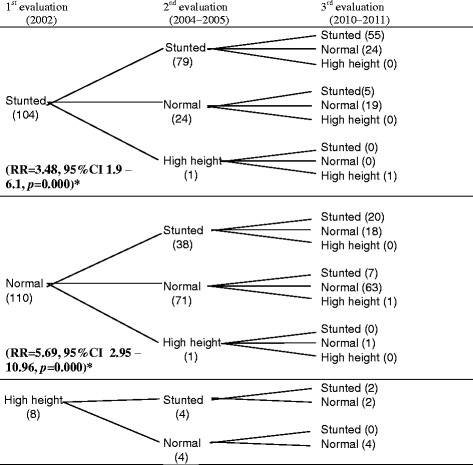
Evolution of the movement of the prospective cohort of children in the different categories of nutritional status

Stunted: <−2SD, normal: −2 to +1DS, high height: >+1 SD (WHO, 2006)

*To calculate the RRs of stunted children of each cohort, stunted children in the 2nd evaluation (2004–2005) were used as exposed group and normal + high height as non-exposed group. Stunted children in the 3rd evaluation (2010–2011) were defined as cases and normal children + high height as non-cases

As far as the risk analysis is concerned, we observed that children who were stunted and did not recover for the second evaluation had 3.4 times higher risk of being stunted in the third evaluation, than children who were firstly diagnosed as stunted but they recovered in the second evaluation, whereas children, who started with a normal nutritional status and in the second evaluation were diagnosed as stunted, had a higher risk of 5.7 times of being stunted in the third evaluation than children who remained with a normal nutritional status in the second evaluation. Following this analysis, the RR was stratified for sex and age (Tables 3 and 4), finding that the statistical significance of the pattern of risk shown in Table 4 did not disappear.

**Table 3:**
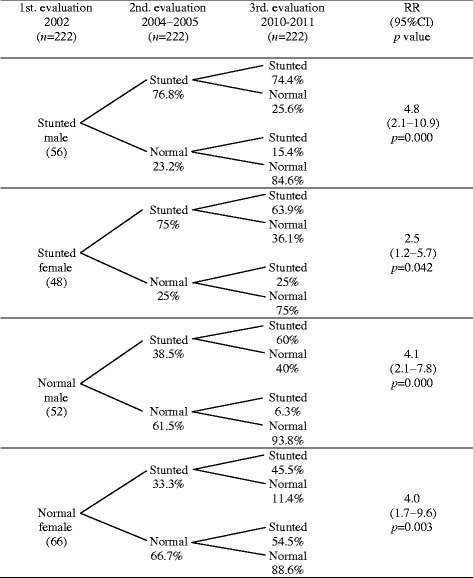
Risk analysis of the children of the cohort according to the height for age index by sex

Stunted: <−2SD, normal: −2 to +1SD and >+1 SD (WHO, 2006). To calculate RR, stunted children were used as exposed group and normal children as non-exposed group (2nd evaluation 2004–2005). Stunted children were defined as cases and normal children as non-cases (3rd evaluation 2010–2011)

**Table 4:**
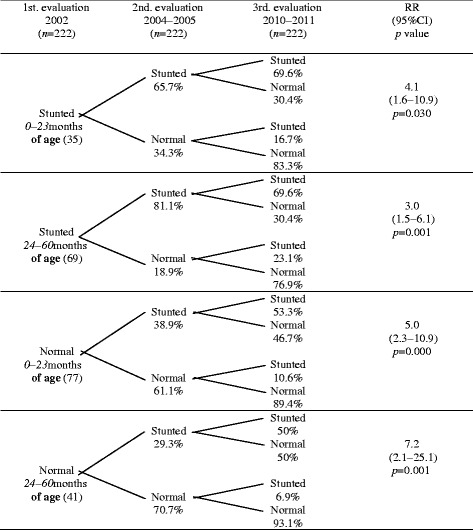
Risk analysis of the children of the cohort according to the height for age index by age

Stunted: <−2SD, normal:−2 to +1SD and >+1 SD (WHO, 2006). To calculate RR, stunted children were used as exposed group and normal children as non-exposed group (2nd evaluation 2004–2005). Stunted children were defined as cases and normal children as non-cases (3rd evaluation 2010–2011)

## Discussion

The purpose of this study was to identify the changes in the nutritional status of a cohort of children of rural communities of Chiapas beneficiaries of Oportunidades on a 9-year follow-up period. Specifically, we were interested in determining the risk for children to continue with their nutritional status diagnosed at the baseline. The main finding of this study was that children who did not recover nutritionally before 3 years of age have a higher risk of continuing with the problem of stunting regardless their gender. While children who were diagnosed at the baseline evaluation as normal, but identified as stunted between 3 and 5 years of age, were at greater risk of continuing with this nutritional condition at school age and adolescence, compared with those who remained normal in the second evaluation or were stunted at the baseline evaluation but recovered in the second evaluation. In spite of the benefits of the Oportunidades program, the population is still at risk of continuing with the vicious circle of the malnutrition. The studied children who in the first evaluation were diagnosed with stunting problems (69.6 %) continued with this health problem after almost 10 years from the first evaluation.

These findings leave us the question, why has not the nutritional status of children improved? A possible answer might be the low effectiveness of Oportunidades in improving the quality of the diet [21] together with the lack of high-quality nutritional surveillance and orientation. As shown in another component of our research, yet unpublished, where we assessed the children’s diet and their families’ diet habits, children are consuming a hypercaloric diet (mainly carbohydrate) with an increase of the industrialized food consumption and a decrease of fruits and vegetables consumption during the follow-up period.

Nevertheless, we recognize the limitation of not having a group of children without the benefits of Oportunidades, due to all families in these communities being included in the Oportunidades program.

Stunting during the first 3 years of life is a negative promoter for a good economic situation in the adulthood; by contrast, an adequate nutrition in the first 2 years of life is essential for the formation of human capital [22, 23]. Chronic malnutrition that occurs in the first years of life causes a shorter size, which explains the smaller size of individuals in developing countries [1, 2]. Several longitudinal studies have shown that the nutritional status of children under 3 years of age determine their adult nutritional status [24–27]. Families of the children in this research have lived in highly marginalized conditions for a long time. Therefore, the short stature of children might be associated to an intergenerational nutritional factor [28]. The intergenerational cycle of growth failure has been described in many developing countries, that is, girls who were stunted in early childhood became stunted women and are more likely to give birth to low birth-weight children [29]. Muzzo suggests that the height of the mother is strongly associated with the height of their children, rather than the height of the father is. There might have a genetic factor that influences growth; however, the extent of the genetic potential might be affected by the socioeconomic and environmental conditions of the children [30].

Our results suggest that the study children might be under the so-called “the double burden of malnutrition transition” [31–33], which implies the coexistence of malnourished children and adults with overweight and obesity within the same families due to the presence of stunting. This situation has been observed in Mexican rural communities of the south of the country [34–36]. During the period of this research, significant changes were observed in the living conditions of the families; it is remarkable to note the increase in the literacy levels and the higher number of assets and better housing conditions of these families. For instance, these families increased their income in $22.94 USD per person in average during the last evaluation (from $6.9 in 2002–2003 to $29.38 USD in 2010–2011). Although their living conditions have significantly improved, these improvements are not reflected in a better nutritional status of their children. This might be the result of an adaptation process achieved through a decrease of growth velocity. The more severe and the longer the malnutrition is, the greater the negative effects on all body measurements are [37, 38]. The results of this research agree with the ones presented in the cited study.

## Conclusions

From the findings of this study, we can conclude that despite the studied population has being exposed to the benefits of Oportunidades during a long time, the problem of stunting persists and affects mostly children who are between 3 and 7 years of age. Therefore, it might be expected that a great proportion of these children will be overweight or obese in their adulthood due to their halted growth and a short stature, which in its turn determines that their weight becomes greater than their size. Thus, it is important to make efforts to watch the growth of the children during their first 3 years of age, to focus on improving the diet of women at fertile age and pay special attention to environmental conditions to break the vicious cycle of malnutrition.

## References

[1] Food and Agriculture Organization of the United Nations. The state of food and agriculture, 2013. Food Systems for Better Nutrition. Online. Retrieved from http://www.fao.org/docrep/018/i3300e/i3300e00.htm. On 15th August 2013.

[2] Fondo de la Naciones Unidas para la Infancia . Informe Annual 2010. Online. Retrieved from: http://www.unicef.org/mexico/spanish/informeUNICEF2010_final_baja.pdf. On 10th September 2013.

[3] Rivera J, Cuevas L, González T, Shamah T, García R (2013). Stunting in Mexico in the last quarter century: analysis of four national surveys. Salud Publica Mex.

[4] Ávila A, Shamah T, Galindo C, Rodríguez G, Barragán LM (1998). La desnutrición infantil en el medio rural mexicano. Salud Publica Mex.

[5] Instituto Nacional de Salud Pública. Encuesta Nacional de Salud y Nutrición 2012. Cuernavaca, Morelos, México:INS,2012;147–154.

[6] Consejo Nacional de Evaluación de la Política de Desarrollo Social. Informe de Evaluación de la Política de Desarrollo Social en México, México D.F: CONEVAL 2012; 28–47.

[7] Instituto Nacional de Salud Pública (2012). Encuesta Nacional de Salud y Nutrición 2012. Resultados por entidad federativa.

[8] Chávez A, De Chávez M, Roldán A, Bermejo S, Avila A, Madrigal H (1996). The food and nutrition situation in Mexico: a food consumption, nutritional status and applied programs tendencies report from 1960 to 1990.

[9] Levy S (2006). Progress against poverty: sustaining Mexico’s *Progresa-Oportunidades Program*.

[10] World Bank. Shanghai poverty conference: case study summary. Retrieved from: Julio 2014 http://web.worldbank.org/archive/website00819C/WEB/PDF/CASE_-62.PDF.

[11] Instituto Nacional de Estadística y Geografía. Panorama Sociodemográfico de Chiapas, México. INEGI 2012.

[12] Consejo Nacional de Población. Chiapas, región norte. Grado de marginación por municipio, 2010. Retrieved from: Julio 2014 http://cuentame.inegi.org.mx/monografias/informacion/chis/default.aspx?tema=me&e=07

[13] World Health Organization (1995). Physical status: the use and interpretation of anthropometry. Report of a WHO Expert Committee.

[14] Lohman T, Roche A, Martorell R (1988). Standardization reference manual.

[15] Habitch (1974). Standardization of anthropometric methods in the field. PHAO Bull.

[16] Sguassero Y, Moyano C, Aronna LA, Fain H, Orellano A, Carroli B (2008). Validación clínica de los nuevos estándares de crecimiento de la OMS: análisis de los resultados antropométricos en niños de 0 a 5 años de la ciudad de Rosario, Argentina. Arch argent pediatr [online].

[17] Peláez ML, Torre P, Ysunza A (1993). Elementos prácticos para el diagnóstico de la desnutrición. Centro de capacitación integral para promotores comunitarios.

[18] World Health Organization, World Health Organization. Multicentre Growth Reference Study Group (2006). WHO child growth standards: length/height-for-age, weight-for-age, weight-for-length, weight-for-height and body mass index-for-age: methods and development.

[19] World Health Organization (2010). Anthro for personal computers, version 3.1: software for assessing growth and development of the world’s children.

[20] World Health Organization (2009). AnthroPlus for personal computers, version 1.0.2: Software for assessing growth of the world’s children and adolescents.

[21] Ramirez-Silva I, Rivera JA, Leroy JL, Neufeld LM (2013). The Oportunidades programs fortified food supplement, but not improvements in the home diet, increased the intake of key micronutrients in rural Mexican children aged 12–59 months. J Nutr.

[22] Martorell R, Melgar P, Maluccio J, Ayreh D, Rivera J (2010). The nutrition intervention improved adult human capital and economic productivity. J Nutr.

[23] Victora C, Adair L, Fall C, Hallal P, Martorell R, Richter L (2008). Maternal and child undernutrition: consequences for adult health and human capital. Lancet.

[24] Victora C, de Onis M, Hallal P, Blossner M, Shrimpton R (2010). Worldwide timing of growth faltering: revisiting implications for interventions. Pediatrics.

[25] Prader A, Largo R, Molinari L, Issler C (1995). Physical growth of Swiss children from birth to 20 years of age. First Zurich longitudinal study of growth and development. Helvetica Paediatrica Acta.

[26] Roche A, Wainer H, Thissen D (1975). Predicting adult stature for individuals. Monographs in paediatrics.

[27] Tanner J, Goldstein H, Whitehouse R (1970). Standards for children’s height at ages 2–9 years allowing for height of parents. Arch Dis Child.

[28] Emanuel I, Kimpo C, Moceri V (2004). The association of grandmaternal and maternal factors with maternal adult stature. Int J Epidemiol.

[29] UNICEF (1998). Estado Mundial de la Infancia.

[30] Muzzo B (2003). Crecimiento normal y patológico del niño y del adolescente. Rev Chil Nutr.

[31] Leroy JL, Habicht JP, González de Cossío T, Ruel MT (2014). Maternal education mitigates the negative effects of higher income on the double burden of child stunting and maternal overweight in rural Mexico. J Nutr.

[32] Ihab AN, Rohana AJ, Manan WMW, Suriati WNW, Zalilah MS, Rusli AM (2013). The coexistence of dual form of malnutrition in a sample of rural Malaysia. Int J Prev Med.

[33] Doak C, Adair L, Bentley M, Monteiro C, Popkin B (2005). The dual burden household and the nutrition transition paradox. Int J Obes.

[34] Gurri FD. La doble carga de la transición nutrimental en zonas rurales de la Península de Yucatán, ¿consecuencia de la alteración de los sistemas agrícolas de subsistencia tradicionales en la segunda mitad del siglo XX? En: Muñoz Cano JM. Obesidad: Problema Multifactorial. (Coord), septiembre 2011:65–84.

[35] Arroyo P, Fernandez V, Loria A, Pardio J, Laviaga H, Vargas-Ancona L (2007). Obesity, body morphology, and blood pressure in urban and rural population groups of Yucatan. Salud Publica de Mex.

[36] Malina R, Peña M, Tan S, Buschang P, Little B (2007). Overweight and obesity in rural Amerindian population in Oaxaca, southern Mexico, 1968–2000. Am J Hum Biol.

[37] Restrepo BN, Restrepo MT, Beltrán JC, Rodríguez M, Ramírez RE (2006). Estado nutricional de niños y niñas indígenas de hasta seis años de edad en el resguardo Embera-Katío, Tierralta, Córdoba, Colombia. Biomédica [revista en la Internet].

[38] Svedberg P. Poverty and undernutrition: theory, measurement and policy. New York: United Nations Universuty (UNU/WIDER) Oxford University Press; 2000.

